# Clinical significance of ^18^F-FDG PET/CT imaging in 32 cases of gastrointestinal stromal tumors

**DOI:** 10.1186/s40001-022-00806-9

**Published:** 2022-09-16

**Authors:** Wen Du, Guojin Cui, Kaiping Wang, Shaojie Li

**Affiliations:** Department of Nuclear Medicine, Da Qing Oilfield General Hospital, No.9 Zhongkang street, Daqing City, 163001 Heilongjiang China

**Keywords:** ^18^F-FDG metabolic imaging, Gastrointestinal stromal tumor, Retrospective study, Tumor, Glucose metabolism level

## Abstract

**Objectives:**

To explore the clinical significance of ^18^F-FDG metabolic imaging in the diagnosis and biological risk assessment of gastrointestinal stromal tumors (GIST).

**Methods:**

This study is a clinical retrospective study. The research subjects were patients with GIST who were admitted to our hospital from January 2014 to December 2019 and underwent ^18^F-FDG metabolic imaging, and the relationship between biological risk and FDG metabolism was analyzed retrospectively.

**Results:**

A total of 32 patients with GIST were included in this study, of which 17 patients had very low and low-risk lesions, and the FDG metabolism level did not increase; five patients had moderate-risk gastric lesions, and the FDG metabolism level was abnormally increased; 10 patients had high-risk lesions, and except for one patient with multiple lesions, the FDG metabolism level of these patients was increased.

**Conclusions:**

The level of glucose metabolism is abnormally increased in tumor cells with vigorous mitosis and has higher biological risk. The ^18^F-FDG metabolism level can determine the biological risk of GIST and whether high-risk lesions involve other tissues and organs, as it more comprehensively reflects the distribution of lesions, the activity of tumor cells and the stage of the disease.

## Introduction

Gastrointestinal stromal tumor (GIST) is a mesenchymal tumor of the gastrointestinal tract. The pathological manifestations are spindle cells or epithelioid cells, and the histochemical characteristics are that the tumor is CD117, CD34, and DOG1 positive [1–3]. Due to the diversity and complexity of the biological behavior of GIST, it cannot be classified solely as benign or malignant; the risk of GIST biological behavior can be quickly and effectively judged by examining the patient's treatment plan and prognosis, as it is closely related to these [4, 5]. ^18^F-FDG metabolic imaging reflects the level of glucose metabolism of tumors, which is positively correlated with the risk of the tumors [6, 7].

At present, the clinical significance of ^18^F-FDG metabolic imaging in the diagnosis and biological risk assessment of GIST is not clear. In this study, the subjects were patients with GIST who were admitted to our hospital and underwent ^18^F-FDG metabolic imaging, and the clinical value of ^18^F-FDG metabolic imaging in the diagnosis and biological risk assessment of GIST was investigated.

## Materials and methods

### Research objects

From January 2014 to December 2019, patients in our hospital with abdominal discomfort, abdominal mass and gastrointestinal tract space occupying lesions found upon physical examination were examined by positron emission tomography/computed tomography (PET/CT), and GIST was finally confirmed by pathology and clinical examination. Of all the patients who participated in the retrospective investigation, 15 were excluded based on the inclusion and exclusion criteria. A total of 32 patients with GIST were included in this retrospective study to confirm the relationship between the tumor’s biological risk and tumor FDG metabolism.

The study complies with the ‘Declaration of Helsinki of the World Medical Association’. Since this study does not require any patient-related interventions or experiments, it was exempted from requiring informed consent after review by the ethics committee of the hospital.

### Inclusion and exclusion criteria

Inclusion criteria: (1) age over 18 years; (2) patients with a clear diagnosis of GIST; (3) patients with GISTs having untreated neurologic metastases or unstable central nervous system metastases. Exclusion criteria: (1) patients with type I insulin-dependent diabetes, poorly controlled type II insulin-independent diabetes or a fasting blood glucose of  > 10 mmol/L (200 mg/dL); (2) patients who had previously undergone chemotherapy or radiation therapy or had a second type of primary tumor; (3) patients having a PET-negative GIST during the baseline examination; (4) patients with renal failure; (5) patients who were allergic to contrast agents; (6) patients with incomplete case data.

### GIST diagnostic criteria

According to the modified GIST standard of the National Institutes of Health (NIH), tumors can be classified as: (1) very low risk: tumor diameter  ≤ 2 cm, mitotic figures  < 5/50 HPF; (2) low risk: tumor diameter 2–5 cm, mitotic figures < 5/50 HPF; (3) moderate risk: tumor diameter  < 5 cm, mitotic figures. 6–10/50 HPF; tumor diameter 5.1–10 cm, mitotic figures  < 5/50 HPF; (4) high risk: tumor rupture; tumor diameter  ≥ 5 cm, mitotic figures  > 10/50 HPF; mitotic figures of any size  > 10/50 HPF; tumor diameter r  ≥ 10 cm, mitotic figures of any size; non-gastric tumors: tumor diameter 2–5 cm, mitotic figures. 6–10/50 HPF; tumor diameter 5.1–10 cm, mitotic figures < 5/50 HPF.

### ^18^F-FDG metabolic imaging

In this study, the ^18^F-FDG metabolic imaging device used was the Philips GIMI 16PET/CT. All patients had been fasting for more than 6 h. Before injection of an imaging agent, 1200 mL of gastrointestinal contrast agent was given orally, then ^18^F-FDG was injected intravenously at rest, at 5–7 MBq/kg body weight; 1 h later, 600 mL of water was taken orally and then the patient was examined on the machine. Three-dimensional acquisition and CT scan were performed from the top of the head to the upper thigh for 2 min per bed.

After the completion of image processing, the range, number and size of the lesions were measured, and the results were used to determine the criteria. According to the tumor diameter, the lesions were divided into a  ≤ 2 cm group, 2–4.9 cm group,  ≥ 5 cm group and  ≥ 10 cm group; the ^18^F-FDG metabolic standardised uptake values (represented as SUVmax) of the lesions were measured separately, and the clinical manifestations, pathological stages and disease development were retrospectively analyzed.

### Statistical analysis

In this study, SPSS 20.0 statistical software was used for data processing. Measurement data are expressed as mean ± standard deviation (x ± s), and counting data are expressed as a percentage (%). *P* < 0.05 indicates that the difference is statistically significant.

## Results

### General information

A total of 32 patients with GIST were included in this study, including 23 males and 9 females, aged 37–68 years. All patients had an even distribution of gastrointestinal contrast agent, good filling of the stomach, clear visibility of the gastric wall and a clear course of the intestinal tract, and the fusion of CT and PET images was accurate, as shown in Table 1.

**Table 1:** Number of lesions in 32 patients with GIST

Position	Number of lesions	*n*	Size of the lesions (diameter)			
			< 2 cm	2–5 cm	5–10 cm	> 10 cm
Stomach	Single lesion	19	6	9	3	1
	Multiple lesions	3		2	1	
Jejunum and ileum	Single lesion	4	2	2		
	Multiple lesions	3		3		
Mesenteric lesion	Single lesion					
	Multiple lesions	3	1	2		
Colon lesion	Single lesion	3		3		
	Multiple lesions	1		1		
Liver	Single lesion					
	Multiple lesions	3	1	1	1	
Abdominal and pelvic lymph nodes	Single lesion					
	Multiple lesions	1		1		
Total	Patients with single lesion	26				
	Patients with multiple lesions	6				

### ^18^F-FDG metabolic imaging results

In the 32 patients with GIST, 26 had a single lesion and six had multiple lesions; 19 cases of single lesions occurred in the stomach, four in the jejunum and ileum and three in the colon. There were 14 lesions in the six patients with multiple lesions, including three cases in the stomach, three in the liver, three in the jejunum and ileum, three cases of mesenteric lesion, one case of colon lesion and one case of abdominal and pelvic lymph nodes (Table 1). The growth of lesions could be divided into three forms: intracavitary growth, extra-activity growth and transmural growth. Most of the lesions appeared as round soft tissue nodules or mass shadows, some of the lesions had leaf-like changes, some had less uniform internal density, and punctiform and flaky high-density shadow and patchy low-density shadow were seen locally.

### GIST risk analysis

Among the 26 cases of GIST with a single lesion, 17 cases were diagnosed by pathological and clinical examination as very low risk and low-risk lesions, five cases were moderate risk and four cases were high risk. Among the 17 cases of very low risk and low-risk lesions, 12 cases were gastric tumors, and the lesion sizes were less than 5 cm; three cases were jejunum and ileum tumors, and the lesion sizes were 2.3–3.0 cm; two cases were colon tumors, and the lesion sizes were 3.0 cm and 4.1 cm (Table 2). ^18^F-FDG imaging of lesions in all 17 patients showed no increase in glucose metabolism. Five cases of gastric tumors were moderate-risk lesions: the size of the lesion was 2.8–6.4 cm, the glucose metabolism level was increased to varying degrees and the SUVmax was 2.3–3.6. Of the four cases of high-risk lesions, two cases were stomach tumors, one case was a jejunum and ileum tumor and one case was a colon tumor. The two cases of gastric tumor lesions were 3.6 cm and 12.7 cm in size, the glucose metabolism level was increased and the SUVmax was 4.4 and 7.7, respectively. The case of the jejunum and ileum tumor was 4.2 cm in size, the glucose metabolism level was increased and the SUVmax was 3.8. The case of the colon tumor was 4.2 cm in size, the glucose metabolism level was increased and the SUVmax was 6.8.

**Table 2:** Distribution of lesions in patients with increased glucose metabolism levels to varying degrees

Position	Single lesion group		
	Very low risk and low risk	Moderate risk	High risk
Stomach	12	5	2
Jejunum and ileum	3		1
Colon lesion	2		1
Total	17	5	4

Among the six cases of GIST with multiple lesions, after pathological examination, the mitosis met the diagnostic criteria for high-risk lesions. Two patients with the stomach and liver involved underwent gastroscopic biopsy to obtain pathological specimens. The gastric lesions were 3.5 and 7.9 cm in size, and the liver lesions were 1.8–4.0 cm in size; all showed an increased glucose metabolism level, and the SUVmax was 4.2–15.6. Two patients with jejunum, ileum and mesenteric lesions underwent laparotomy and pathological examination. The jejunum and ileum lesions were 3.7 cm and 4.7 cm in size, the mesenteric lesions were 2.0–3.4 cm in size, the glucose metabolism level was abnormally increased and the SUVmax was 3.4–8.1. One patient with gastric, liver, and jejunum and ileum lesions underwent gastroscopic biopsy. The gastric lesion was 6.3 cm in size, and the remaining lesions were 1.6–3.3 cm in size; the glucose metabolism level of all the lesions was increased, and the SUVmax was 3.1–9.8. A patient with colon, jejunum, ileum, mesenteric and retroperitoneal lymph node lesions in the abdominal cavity underwent colonoscopic biopsy. Except for one mesenteric lesion of 1.1 cm, there was no increase in glucose metabolism; the glucose metabolism of the remaining lesions was increased. The colon lesion was 4.3 cm in size, the remaining lesions were 2.1–3.6 cm in size, and the SUVmax was 3.0–4.6. See Table 3 for details. These results indicate that the level of glucose metabolism of tumor cells with vigorous mitosis is abnormally increased and creates a higher biological risk.

**Table 3:** Correlation between degrees of lesions and glucose metabolism levels in patients with GIST

Glucose metabolism levels	Single lesion group			Multiple lesion group
	Very low risk and low risk (*n* = 17)	Moderate risk (*n* = 5)	High risk (*n* = 4)	High risk (*n* = 6)
SUV max	1.34 ± 0.43 (0.63–2.68)	2.75 ± 0.36 (2.3–3.6)	5.68 ± 1.87 (0.36.3.8–6.8)	7.95 ± 2.43 (3.1–15.6)
*P* value	0.0154			< 0.001

## Discussion

A total of 32 patients with GIST were included in this study, of which 17 had very low and low-risk lesions, and the FDG metabolism level did not increase in these patients. Five patients had moderate-risk gastric lesions with abnormally increased FDG metabolism. Ten patients had high-risk lesions; the FDG metabolism level in these patients increased, except in one patient with multiple lesions.

Gastrointestinal stromal tumors are the most common mesenchymal tumors originating from the gastrointestinal tract. They have malignant potential, and it is widely accepted that a diameter of GIST  ≥ 20 mm should be treated [8]. The diagnosis of GIST originates from the development of molecular biology, and needs to be determined by combining histological findings and immunohistochemical results [9, 10]. The biological risk of GIST is closely related to the location, size, shape, density, presence or absence of necrotic cystic degeneration and calcification, presence or absence of ulceration, invasive growth and the blood supply of the tumor; but the most critical factor in determining the biological risk of GIST is its histological mitotic number, which is the proliferation state and activity of the cell.

Accurate identification of chemically refractory sites of GIST is very important for the treatment of drug-resistant tumors. Additional imaging techniques (such as brain CT scans or magnetic resonance imaging [MRI] scans) have been proposed to monitor disease and assess recurrence in high-risk patients. Among them, ^18^F-FDG PET/CT is a non-invasive method to study biochemical and metabolic changes in tumor tissues, which provides useful functional information for identifying surviving tumor tissues [11]. ^18^F-FDG metabolic imaging expresses the metabolic level of glucose molecules in the cell [12–14]. Tumor cells have increased nucleus division, active cell proliferation, and increased glucose metabolism, which is manifested by increased FDG metabolism and an increased SUV at the lesion. The indicators of the active degree of tumor cell proliferation, that is, the number of nucleus divisions and the glucose metabolism level of the lesion, are positively correlated, and the degree of risk is proportional to the SUV of the lesion [15, 16].

When ^18^F-FDG metabolic imaging was performed in patients with GIST, 18 of the 32 patients had normal glucose metabolism levels; all of these patients had single lesions, seven cases with a lesion size less than 2 cm, 10 cases with lesions 2–5 cm and one case with a gastric lesion of 5.4 cm. In the 17 cases with lesions smaller than 5 cm, ^18^F-FDG metabolic imaging showed no abnormal increase in glucose metabolism. Combined with histological examination, GIST with a mitosis < 5/50 HPF were diagnosed as very low and low risk. The one case of a single gastric lesion of 5.4 cm showed no increase in glucose metabolism level, and histological examination showed mitosis  < 5/50HPF. The NIH diagnostic criteria classify gastric GIST of more than 5.0 cm with a mitosis less than 5/50 HPF as moderate-risk lesions, so we can judge that the biological risk of the tumor is medium risk by the tumor diameter, but the mitosis of the tumor cells is not active, that is, the level of glucose metabolism does not increase.

Among the 32 patients, ^18^F-FDG metabolic imaging of 14 cases showed elevated glucose metabolism levels in the lesions, of which eight cases were single lesions and six cases were gastric lesions. One case of gastric lesion with a diameter greater than 10 cm was directly classified as a high-risk lesion under the NIH diagnostic criteria; five cases of 2–10 cm lesions that occurred in the stomach, and four other cases were diagnosed as moderate-risk lesions by histological examination. Another patient with a 3.6 cm gastric lesion had a glucose metabolism SUV of 4.4, and histological examination of mitosis  > 10/50 HPF, which was diagnosed as a high-risk lesion. Therefore, for gastric lesions of 2–10 cm in size with increased glucose metabolism, it is difficult to use ^18^F-FDG metabolic imaging alone to define the moderate and high biological risk of the lesion. Among the eight single lesions, one case was a jejunum and ileum lesion and one case was a colon lesion: the sizes were 2.7 cm and 3.5 cm, respectively; the glucose metabolism was increased in both, and the SUVmax was 4.1 and 6.9, respectively.

In the six cases with multiple lesions, 13 of the 14 lesions had elevated glucose metabolism levels. The malignant indicators of GIST included metastasis and invasion of other tissues and organs. The final histological examination showed that the cells were active in mitosis. ^18^F-FDG metabolic imaging showed that the glucose metabolism of the tumor cells was active. In one case of jejunum and ileum lesion with a mesenteric lesion of 1.1 cm in size, there was no increase in glucose metabolism in the mesenteric lesion; the tumor diameter in the jejunum and ileum was 3.7 cm, the glucose metabolism level was increased and the SUVmax was 4.9. After exploratory laparotomy, histological examination showed the two lesions were GIST; combining the histology and the characteristics of multiple tumor lesions, the clinical diagnosis was that of high-risk lesions.

According to the NIH diagnostic criteria of GIST in 2019, tumor diameters  < 2 cm are classified as very low-risk lesions with mitotic figures  < 5/50 HPF, and mitotic figures  > 10/50 HPF are high-risk lesions. In this study, among the 32 patients, six of the single lesions with tumor diameter less than 2 cm had normal glucose metabolism level and were diagnosed as very low risk lesions by pathological and histological examination, with mitotic figures  < 5/50 HPF. Three of the multiple lesions were less than 2 cm in diameter, but they were classified as high-risk lesions due to metastasis and invasive growth. FDG metabolic imaging showed that FDG metabolism was higher in all the lesions except in one negative lesion.

There were 19 cases of patients with tumor diameters of 2–5 cm; there were 16 cases of a single lesion, 10 cases with mitotic figures  < 5/50 HPF, as well as seven cases of stomach and three cases of non-gastric lesions with normal glucose metabolism that were all classified as low-risk lesions; four cases of gastric lesions with 6–10 mitotic figures per 50 HPF were moderate-risk lesions. The glucose metabolism level was increased to varying degrees, which was significantly different from low-risk gastric lesions, but not significantly different from high-risk gastric lesions of the same size; one case of gastric lesion and one case of colon lesion had abnormally increased glucose metabolism with mitotic figures  > 10/50 HPF, as shown in high-risk lesions. In a single lesion with a size of 2–5 cm, whether the glucose metabolism level is normal or not is the standard for distinguishing low and medium–high risk; it is also the standard for distinguishing low- and high-risk non-gastric lesions, but for moderate-risk lesions in the stomach, glucose metabolism level alone is not enough to distinguish between moderate and high risk. Compared with non-gastric lesions, the pathology of gastric lesions is easier to obtain. For those in the jejunum and ileum, mesentery and colon, where pathological specimens are not easy to obtain, ^18^F-FDG metabolic imaging of lesions is more meaningful for the differentiation of risk. The remaining three cases had multiple lesions of 3.5 cm in the gastric lesion, 3.7 cm in the jejunum and ileum lesion and 4.3 cm in the colon lesion, and the glucose metabolism increased. The clinical manifestation, histological examination and glucose metabolism level all supported highly malignant lesions.

Among the patients with tumor diameters of 5–10 cm, there were four cases of elevated glucose metabolism: three cases of single gastric lesions were moderate-risk lesions, and one case of multiple gastric lesions had high-risk lesions. One case with a tumor diameter greater than 10 cm had elevated glucose metabolism and was diagnosed as a high-risk disease according to the NIH diagnostic criteria.

In summary, for GIST with diverse and complex biological behaviors, the NIH diagnostic criteria directly classifies tumors larger than 10 cm in diameter, non-gastric lesions larger than 5 cm and multiple lesions as high-risk lesions. The level of glucose metabolism of tumor cells revealed by ^18^F-FDG metabolic imaging is positively correlated with the mitotic activity of tumor cells [17–20]. For lesions whose biological risk cannot be judged solely by the size of the tumor, if the diameter of a single tumor is less than 5 cm and the glucose metabolism level of ^18^F-FDG metabolic imaging is normal, they are considered to be GIST lesions of very low or low risk. For a single lesion of 2–10 cm in the stomach with an elevated glucose metabolism level, there are two possible clinical possibilities: moderate risk and high risk; a tumor with a diameter of 5–10 cm and a normal glucose metabolism level is a moderate risk lesion. Non-gastric lesions with a tumor diameter of less than 5 cm and increased glucose metabolism level are considered to be high-risk lesions. For GIST with multiple lesions, ^18^F-FDG metabolic imaging can more easily find small lesions with an increased glucose metabolism level, and for all lesions with an increased glucose metabolism level, the SUV value can be used as a quantitative parameter of tumor cell activity before and after treatment to evaluate the therapeutic effect [21, 22].

There are some deficiencies in this study. First, this study is a retrospective study without a control group, so there is a certain risk of bias. Second, this study is a single-center clinical study, and a multi-center clinical study is needed to allow for further discussion. Finally, the sample size included in this study is relatively small, and it is necessary to increase the sample size in further research.

## Conclusions

The biological risk of GIST is closely related to cell mitosis, and the level of glucose metabolism is abnormally increased in tumor cells with vigorous mitosis. When the biological risk cannot be judged by tumor size and shape, the ^18^F-FDG metabolic level can be used to determine the biological risk of GIST and whether high-risk lesions involve other tissues and organs, as it more comprehensively reflects the distribution of lesions, the activity of tumor cells and the stage of the disease.

## Data Availability

All data generated or analyzed during this study are included in this published article.
